# Valuing the Economic Costs of Allergic Rhinitis, Acute Bronchitis, and Asthma from Exposure to Indoor Dampness and Mold in the US

**DOI:** 10.1155/2016/2386596

**Published:** 2016-05-29

**Authors:** David H. Mudarri

**Affiliations:** The Cadmus Group, Inc., 2909 Pierpont St, Alexandria, VA 22302, USA

## Abstract

Two foundational methods for estimating the total economic burden of disease are cost of illness (COI) and willingness to pay (WTP). WTP measures the full cost to society, but WTP estimates are difficult to compute and rarely available. COI methods are more often used but less likely to reflect full costs. This paper attempts to estimate the full economic cost (2014$) of illnesses resulting from exposure to dampness and mold using COI methods and WTP where the data is available. A limited sensitivity analysis of alternative methods and assumptions demonstrates a wide potential range of estimates. In the final estimates, the total annual cost to society attributable to dampness and mold is estimated to be $3.7 (2.3–4.7) billion for allergic rhinitis, $1.9 (1.1–2.3) billion for acute bronchitis, $15.1 (9.4–20.6) billion for asthma morbidity, and $1.7 (0.4–4.5) billion for asthma mortality. The corresponding costs from all causes, not limited to dampness and mold, using the same approach would be $24.8 billion for allergic rhinitis, $13.5 billion for acute bronchitis, $94.5 billion for asthma morbidity, and $10.8 billion for asthma mortality.

## 1. Introduction

*Overview*. Few scientific studies have addressed the cost and benefit issues of any indoor pollutants quantitatively because of the complexity of quantifying both risks and costs. Yet policy makers increasingly ask for cost information in their effort to develop and prioritize programs for indoor air quality and health. There is growing evidence from the international literature that 15–20% of the economic cost of several diseases and disease symptoms is associated with indoor air exposures to dampness and mold. This study provides estimates of attributed annual costs associated with allergic rhinitis (AR), acute bronchitis, and asthma from such exposure.

The two foundational ways to measure the economic cost of illness are referred to as “cost of illness” (COI) and “willingness to pay” (WTP). The conceptual difference between these methods is important. The COI method measures the cost to society for medications and health care services for treating an illness plus the value of reduced production measured by lost earnings due to illness. It also includes the discounted value of lost earnings due to premature death when mortality is involved. COI is the primary method used by the medical and public health professions and is supported by large databases from surveys of illnesses in the population and medical expenditures. COI estimates may not include values for sick days other than workdays or missed schooldays and may not include equivalent burdens to persons who do not seek medical care or burdens to families and friends of persons that are ill.

Conversely, WTP is the dollar value that would just compensate people for having an illness to the point of being indifferent between the monetary compensation and being ill. WTP is expected to be larger than COI because it implicitly includes all the COI costs plus impacts on others close to the patient, other costs such as transportation, and, importantly, the value of intangibles such as pain and suffering. Conceptually, WTP is preferred as a true cost estimate, but it is more theoretical than practical because of difficulties in estimation.

Historically, WTP values for mortality are more developed than for morbidity. The US Environmental Protection Agency (EPA) estimates the value for each premature death to be just over $8 million (2014$), not accounting for age, health status, or cause of death, though a large degree of uncertainty is associated with this estimate [1]. COI values of premature death are age-adjusted based on the discounted value of the expected life earnings remaining. The expected earnings decrease rapidly with age, especially past retirement. Ideally, an age-adjusted WTP value would be preferred, but the evidence of the effect of age is mixed and judged to be insufficient at this time to include in cost benefit valuations [1].

WTP estimates for morbidity are mostly based on a structured questionnaire, and while WTP values are available for some illness symptoms, results may be unique to the characteristics of those interviewed, including their health status and health experience, and therefore are only approximately applicable across diseases. Thus, COI values are often used as a more practical alternative.

Increased hospitalization is closely related to increases in outdoor air pollution exposure and is a major health endpoint when estimating health related economic costs of outdoor air pollution. In examining such costs, Thayer et al. [2] have demonstrated that COI estimates for hospitalization better approximate WTP values if the COI estimates account for many costs that are implicitly included in WTP. For hospitalization, this includes costs to family and friends of the patients and the cost of recovery out of the hospital, which can be significant for respiratory diseases associated with outdoor pollution.

The intent in this paper is to estimate full disease costs attributable to dampness and mold using available COI and WTP estimates to come as close to ideal WTP estimates as possible. The framework for hospitalization used by Thayer is used in this paper to highlight the sensitivity of results to alternative assumptions and methods. For acute bronchitis and asthma, the sensitivity analysis is limited to costs related to hospitalization. The full cost of these diseases, however, is included in the final estimates. Information from the sensitivity analysis is used to estimate the range of costs around the final estimates for all three diseases.

## 2. Estimates of the Health Risk of Dampness and Mold

### 2.1. General Literature Reviews

Substantive literature reviews [3–7] summarize evidence from a large number of studies demonstrating that damp or moldy conditions in buildings pose potentially significant risks for allergenic and infectious diseases. Meta-analyses from this literature are used to quantify the risks for allergic rhinitis, acute bronchitis, and asthma associated with indoor dampness and mold. It should be noted that uncertainty remains about the specific causal agents in damp or moldy environments that result in such illnesses. A summary of these reviews follows.

A review of literature up to 1998 by Bornehag et al. [3] found odds ratios ranging from 1.4 to 2.2 for different health endpoints. However, the potential biases inherent in cross-sectional studies that dominated the literature, as well as the use of varied risk factor metrics (e.g., visible mold, damp stains, condensation, water damage, and damp or mold odor), weakened confidence of causation. Despite these issues, the researchers concluded that the evidence of a causal relationship between dampness and health effects was “strong,” but that evidence of specific causal agents was “inconclusive.”

A 2004 review of studies between 1998 and 2000 by Bornehag et al. [4] found that dampness in buildings is a risk factor for health effects among both atopic and nonatopic individuals and found that dampness approximately doubles the risk for both children and adults. Most of the reviewed studies focused on children, and most were of cross-sectional rather than more robust longitudinal designs. Nevertheless, the authors suggest that causation is indicated since reported associations between objective measures of exposure (e.g., professionally identified) and health are in the same range of associations of more diffuse measures.

An extensive literature review by the Institute of Medicine (IOM) in 2004 [5] evaluated the strength of evidence of relationships between dampness and mold with several health endpoints. They found (a) sufficient evidence for an association of dampness and mold with upper respiratory tract symptoms, wheeze, cough, asthma exacerbation, and hypersensitivity pneumonitis in susceptible persons; (b) limited or suggestive evidence of an association between the presence of mold or other agents in damp environments and lower respiratory illness in otherwise healthy children; (c) sufficient evidence of an association between damp indoor environments and upper respiratory track symptoms, cough, wheeze, and asthma exacerbation; and (d) limited or suggestive evidence of an association between dampness and dyspnea (shortness of breath), lower respiratory illness in otherwise healthy children, and asthma development. However, they found no health outcomes for which there was sufficient evidence of a specific causal agent for dampness or the presence of mold.

A 2011 recent review by Mendell and colleagues [7] combines the findings of the IOM 2004 review with an evaluation of the additional literature up to 2009. They placed increasing weight on studies of stronger designs to account for the relative weakness of cross-sectional studies compared to cohort studies and intervention trials. The highest value was placed on intervention trials. In evaluating the strength of the overall evidence, they considered both the strength of study designs and the number and consistency of results across a wide variety of settings.

Using such criteria, and following the IOM classification of evidence, Mendell et al. [7] found (a) sufficient evidence of an association between dampness or mold with upper respiratory tract infections, cough, wheeze, and asthma exacerbation, including strong suggestive evidence of causality for asthma exacerbation, (b) sufficient evidence for an association between dampness and mold and asthma development, current asthma, ever asthma, dyspnea, respiratory infections, bronchitis, allergic rhinitis, and eczema.

Similar to the other reviews, the authors demonstrated only limited findings of risks for specific health outcomes associated with quantitatively measured microbial factors. They found mixed results from studies using 53 specific types of microbial measures. Suggestive evidence of associations was not found for airborne microbial measures but was found for a limited number of measures in dust. This suggests that exposure to the personal cloud of dust created by a person's movement may be more important than ambient concentrations.

With respect to potential causative agents, suggestive evidence was found for an association between increased concentrations of ergosterol concentrations in dust and current asthma and for an association of higher endotoxin concentrations in dust and increased wheeze. Findings for (1→3)-*B*-D-glucan in dust were mixed showing increases in wheeze for medium concentrations but decreases in wheeze with high concentrations. In addition, no evidence was found of an association with airborne microbial concentrations and health outcomes [7]. Thus, based on research to 2009, qualitative measurements of both dampness and microbial activity such as visible mold, mold odors, or water damage appear to be more reliably associated with health outcomes than quantitative measures of potential causative agents.

### 2.2. Meta-Analyses

The wealth and relative consistency of studies have spawned a number of meta-analyses that provide composite estimates of risk [8–12] of specific health outcomes from exposure to dampness and mold. These estimates are summarized below. Risk estimates are reported as odds ratios or summary effect estimates (CI 95% range):

- Fisk et al. [9] conducted a meta-analysis in 2007 of 33 studies to estimate the added risk of various respiratory health effects associated with exposure to dampness and mold in homes. The health effects studied include upper respiratory symptoms, cough, wheeze, current asthma, ever-diagnosed asthma, and asthma development. Statistically significant estimates ranged from 1.37 (1.23–1.53) for ever-diagnosed asthma for all groups to 1.75 (1.56–1.96) for cough in children.
- Antova et al. [11] conducted a meta-analysis in 2008 of 12 cross-sectional studies on associations between visible mold and several respiratory and allergic health outcomes in children aged 6 to 12. Original studies were from US, Russian, and European households. Significant risks included 1.35 (1.20–1.51) for asthma, 1.38 (1.29–1.47) for bronchitis in the last 12 months, and 1.35 (1.18–1.53) for hay fever. Significant results were also found for various measures of wheeze and cough.
- Fisk et al. [10] conducted a meta-analysis in 2010 using 23 studies on the relationship between dampness and mold and various categories of respiratory infections and bronchitis. Significant risks included 1.45 (1.32–1.59) for bronchitis of all symptom categories, to a range from 1.44 to 1.50 for various measures of respiratory infections for children and adults.
- Quansah et al. [12] conducted a meta-analysis in 2012 using 16 studies of cohort or incident case-control design to study the relationship between measures of dampness or mold and the incidence of asthma. Summary risk estimates ranged from 1.29 (1.04–1.60) for visible mold to 1.73 (1.19–2.50) for mold odor, but the relationship for water damage was not statistically significant.
- Jaakkola et al. [8] conducted a meta-analysis using 31 studies for quantifying the risks posed by dampness and molds for rhinitis, allergic rhinitis (AR), and rhinoconjunctivitis. Risk estimates ranged from 1.51 (1.39–1.64) for rhinitis and visible mold to 2.18 (1.76–2.71) for rhinitis and moldy odor.

The causal agents and mechanism for illness could be complex. For example, dampness can promote mold, bacterial growth, dust mites, and chemical release from building materials. Similarly, molds produce various toxic secondary metabolites (mycotoxins). It is possible that inhalation of these agents, singly or in unknown combinations, creates inflammatory and/or immunosuppression responses that result in the development or exacerbation of respiratory illnesses [5, 6].

Quansah et al. [12] used only studies with cohort or incident case-control designs and their results strengthen the inference of causal effects from dampness and mold. The strength of the association with asthma incidence uniformly increased from dampness, to visible mold, to mold odor. The strong association with mold odor suggests the presence of a biological activity that reaches the breathing zone and implies that the long-term presence of dampness may be necessary to produce such an odor [12]. A similar result was found by Jaakkola et al. [8] in which the strongest relationship was between rhinitis and moldy odor.

While uncertainties remain, these meta-analyses provide the best composite estimate from the literature to date of the health risks associated with exposure to dampness and mold and provide a useful basis for preliminary estimates of disease costs from such exposure.

## 3. Methods

The economic costs of allergic rhinitis, acute bronchitis, and asthma associated with dampness and mold are calculated as the product of the proportion of each disease attributable to dampness and mold (attributable fraction), the estimated disease prevalence in the US, and an estimate of the economic cost of the disease. Attributable fractions and disease prevalence are first established from the estimated risks in the literature and an estimate of prevalence for dampness and mold in the US. An exploration of assumptions and methods of economic valuation and a sensitivity analysis are then conducted to help establish a preferred valuation and potential range of costs. The sensitivity analysis for acute bronchitis and asthma is limited to costs associated with hospitalization and recovery. Final estimates, however, are for the full economic cost for each disease. The sensitivity analysis and other considerations are used to establish the range of costs around the final estimates.

### 3.1. Attributable Fraction and Disease Prevalence

Risk estimates for exposure to dampness and mold were taken only from meta-analyses since those were considered the most representative. Odds ratios and summary effect estimates were considered reasonable approximations of relative risks [9, 13] because the prevalence of the diseases in the US was below 15% for each disease. The fraction of the number of disease cases attributable to exposure to indoor dampness and mold was computed using the following relationship [13]:

(1) $$\begin{array}{c} AF=\frac{\left[P\left(RR-1\right)\right]}{\left[P\left(RR-1\right)+1\right]}, \end{array}$$

where AF is the attributable fraction, *P* is the prevalence of the risk factor (dampness and mold), and RR is the relative risk of exposure (ratio of risk in the exposed population to the unexposed population).

Most of this study focuses on morbidity costs, which include “direct costs” (medications, ambulatory care, hospitalization, etc.) and indirect costs (lost days of work or lowered productivity). Direct costs are not broken down in detail except where necessary for a particular issue raised. Indirect costs are analyzed in more detail to reveal the impact of alternate assumptions on the cost estimates. A set of conservative assumptions is used to calculate base costs. Alternative assumptions for calculating individual cost elements are used in a sensitivity analysis to examine their impact on the total cost estimate.

To calculate AF, the prevalence of dampness and mold indoors for the base case analysis was assumed to be 35%. That is the midrange of approximately 20–50% referenced in other studies [6, 8, 10] that consider many regions in North America and Europe and differs from the 47% prevalence found by Mudarri and Fisk [13]. Both estimates are used in the sensitivity analysis. While health risks for adults and children can differ, health risk estimates for adults was used to represent both adults and children to make this analysis more manageable.

The following meta-analyses described earlier were used in the cost impact calculations:

- Fisk et al. [9]: the estimate of risk for current asthma was 1.56 (1.30–1.86) based on 10 studies.
- Fisk et al. [10]: the risk estimate for bronchitis was 1.45 (1.32–1.59) using 13 studies. The included studies reflect indicators of both acute and chronic bronchitis but are attributed to acute bronchitis for reasons described below.
- Jaakkola et al. [8]: mold odor involved the largest risks for each disease category, which for AR was 1.87 (0.95–3.68). For visible mold, the increased risk for AR was 1.51 (1.39–1.64). The increased risk for visible mold of 1.51 is used to represent the broader category of dampness and mold.

Estimates from Antova et al. [11] were not used due to its sole focus on children, nor were estimates from Quansah et al. [12] because outcomes were for asthma incidence rather than prevalence for which national cost data is more readily adaptable.

Table 1 presents the odds ratios, disease prevalence rates, and attributable fractions used in this study.

The risk estimate in Fisk et al. [10] reflects the effect of a combination of both acute and chronic bronchitis indicators. The number of studies from which this estimate was derived was insufficient for the authors to calculate separate risk estimates. Acute bronchitis is most often viral, while chronic bronchitis is most commonly associated with smoking or chronic exposure to other smoke particles. For this analysis, the odds ratio in Fisk et al. [10] is assumed to be a reasonable estimate of risk for acute bronchitis because the results are consistent with those in Antova et al. [11] for children, who are not likely to have chronic bronchitis but who may have acute bronchitis or bronchiolitis. The latter has similar manifestations common to young children and its inclusion is consistent with the broad based metrics for bronchitis included in the studies that formed the basis for the Fisk et al. [10] meta-analysis. Both of these illnesses are included in the term “acute bronchitis” used in this paper. Exposure to dampness and mold may result in an inflammatory and/or immunosuppressive response [5, 6] that could explain the association to dampness and mold.

### 3.2. Alternative Morbidity Cost Assumptions and Methods

Indirect costs in COI studies include the value of lost workdays and missed schooldays. These are commonly calculated using prevailing wages or incomes, but there is no standard method for choosing such values for nonworking adults or children. Further, reduced productivity while at work as well as the value of pain and suffering is most often not included because of difficulties in quantifying their value. The burden for being sick on nonworkdays or nonschooldays is seldom considered. Except for lost time for parents caring for children during missed schooldays, the costs suffered by family and friends that are engaged in patient support and care are seldom included, as are the full health burdens for persons who underutilize health care services. These are all factors that can affect cost estimates. Studies may also differ in attributing costs to one or another disease because of the complexities of comorbidity between diseases.

In this study, we use COI values exclusively for the base case morbidity costs, selected WTP values as options for sensitivity analysis of morbidity costs, and WTP for the cost of non-hospital-related costs of acute bronchitis and for asthma mortality.

In WTP studies, individual assessments of losses are used to assess what persons are willing to pay to avoid an illness or illness episode. For morbidity costs, this is normally done through a structured questionnaire of subjects. What that covers depends on the nature of the questionnaire. The WTP study by Johnson et al. [16] included participants who were instructed to assume they would be compensated for all medical costs and lost income. This tends to limit their responses to the value of pain and suffering. As is typically done, WTP values in Johnson et al. [16] are scaled by type of symptoms (e.g., nasal and breath), their intensity (e.g., mild and in hospital), length of time (1, 5, or 10 days), and activity restrictions (e.g., need help functioning). These values are included in this present study for hospitalized asthma and bronchitis patients because requisite information on hospitalization and recovery is available. AR symptoms, however, tend to be more continuous over long periods so that available WTP values could not be included for AR.

The value of time lost for workers is normally calculated using the average wage [17, 18]. However, the median wage, which is considerably lower [19], is used in the base case analysis for this study because it is more stable across different occupational subgroups and because the average wage may be unduly influenced by a small percentage of highly paid individuals. The median wage is also used in Thayer et al. [2]. In the base case, time lost to illness for workers is calculated only as workdays lost or as 5/7 (workdays per week) of sick days. For children, schooldays are assumed to be 5 days per week for 9 out of 12 months. All sick days lost for workers and children, including weekend days, are also valued as an option in the sensitivity analysis, though are seldom included in other studies.

There is no standard way of calculating wage-related COI values for nonworkers to account for lost performance of housework, child care, leisure, volunteer activities, and so forth. One could assume that, for adults, these activities are as valuable as working based on expressed preference for these activities over working income. However, limitations to mobility between working and nonworking options might suggest a lesser value. For the base case, a conservative 50% of the median wage is used for nonworking adults and 25% for sick children, for all days lost to illness. The full median wage, the average wage, and valuing all sick days rather than just workdays or schooldays are also used as options in the sensitivity analysis.

For asthma and acute bronchitis for patients who work, the WTP from Johnson et al. [16] can logically be added to COI measures of medical costs and work pay losses to account for pain and suffering. Conceptually, for nonworking adults and children who are sick, a choice could be made between using COI or WTP values but not both. This is because the WTP values in Johnson et al. [16] are limited to only nonincome related burdens. Thus, for nonworking patients, the WTP values should account for all the opportunity costs of illness. Accordingly, for asthma and acute bronchitis hospitalization, WTP and COI methods are treated separately as options for nonworking patients in the sensitivity analysis. For nonworking family and friends providing support, only the COI values are used.

For allergic rhinitis, hospital costs account for only 1% of total medical costs [20] with the remaining costs due to ambulatory care services and high medications costs. Government estimates [20] are used to account for medical costs for allergic rhinitis in the base case, while an alternative measure using incremental medical expenses attributed to allergic rhinitis over nonallergic rhinitis patients, yet controlling for confounders [21] is also used in the sensitivity analysis.

Data are available for allergic rhinitis that allow for calculations of productivity losses from reduced productivity while at work to be included. Bhattacharyya [22] estimated sick workdays for allergic rhinitis adult patients and also estimated odd ratios for days of functional work limitations relative to nonallergic rhinitis patients. Mitchell and Bates [23] provide data on productivity limited days for persons with and without illnesses. These data are used to represent the days of reduced work productivity for the nonallergic rhinitis patients referred to in Bhattacharyya [22] to calculate equivalent days lost for allergic rhinitis patients because of reduced productivity in performing tasks at work or elsewhere.

### 3.3. Incremental Sensitivity Calculations

A sensitivity analysis examines the impact of alternative assumptions of individual cost elements for acute bronchitis and asthma costs related to hospitalization and for the full cost of allergic rhinitis. This is done by incrementally adding WTP values for pain and suffering to working patients, substituting WTP for COI estimates for nonworking patients and children, using full rather than partial pay estimates for nonworkers and children, adding full pay values for nonworkdays and nonschooldays, using the average rather than the median wage rate, and assuming the 47% prevalence of dampness and mold used in Mudarri and Fisk [13]. Finally, for allergic rhinitis, a higher estimate of medical costs provided by Bhattacharyya [21] is also included as an alternate measure for allergic rhinitis costs.

### 3.4. Final Preferred Estimates

Results from the sensitivity analysis are informative but not conclusive. Final preferred estimates must involve reasoned judgments. In this paper, the sensitivity analyses help provide the range of reasonable estimates but the final chosen estimates are not necessarily the midpoint of the range, as is the case in traditional statistical analyses. The final preferred estimate for allergic rhinitis is taken directly from the sensitivity analysis results after accounting for underutilized health care services. Since the sensitivity analysis for acute bronchitis is limited to hospitalization, a WTP estimate from EPA [1] is used for nonhospitalized cases to provide a complete final estimate of costs. For asthma, the sensitivity analysis is also limited to hospitalization. Therefore, the most recent complete morbidity COI estimate of asthma [17] is used as a base estimate and modified as appropriate based on the sensitivity analysis and underutilized health care services. A recent COI estimate for asthma mortality [18] is combined with a WTP estimate of the value of premature death [1] to provide a preferred estimate of the mortality costs of asthma.

### 3.5. Population and Economic Values Used

All data used are adjusted to reflect the 2014 population and prices using the Bureau of Census [24] population figures and the Consumer Price Index (CPI). Wage rate information is from the Bureau of Labor Statistics [19]. The 2014 population, wage, and school data and relationships used are as follows:

 US Population: 318.9 mill:
   workers: 54%,
   nonworkers: 31%,
   children: 15%.
 Med–Av wage/day: $136.72–181.68:
   workdays/weekdays: 0.71,
   schooldays/weekdays: 0.71,
   school wks/annual wks: 0.75.

## 4. Results and Discussion

Results of the sensitivity analysis are presented first, followed by final estimates of the economic cost of each disease.

### 4.1. Results of Sensitivity Analysis

The base case COI costs are established using the median wage or portions thereof as previously described for the time values of workers, nonworking adults, and children. Results are presented first for allergic rhinitis and then for acute bronchitis and asthma hospitalization. For acute bronchitis and asthma, the base case is followed by a presentation of WTP estimates for pain and suffering, including weekend sick days for adult working patients, and all patient sick days for nonworkers and children. All costs presented are those attributable to dampness and mold unless otherwise indicated. All data in Tables 2 and 3 are for base case conditions.

### 4.2. Base Case for Allergic Rhinitis (AR)

#### 4.2.1. Proportion of Allergic Rhinitis due to Dampness and Mold

Calculation of the attributable fraction depends partly on the assumption of the prevalence of dampness and mold. Assuming 35% prevalence for dampness and mold means that 15% of allergic rhinitis cases in the US can be attributed to dampness and mold exposure.

#### 4.2.2. Costs of Allergic Rhinitis

The dominant costs for allergic rhinitis are direct medical expenses for ambulatory care and medication. Indirect (wage-related) costs include lost productivity from both sick days out of work or at home and reduced productivity days while working or performing other tasks.  Table 2 presents the results that are explained below.

#### 4.2.3. Medical Expenses

Approximately 3.59 million cases of AR are attributable to dampness and mold. The US government estimate [20] of the cost of ambulatory care and medication for AR adult patients is $629.69 (inflation adjusted) per patient, most of which is for medications. Assuming the same expenses for children, that amounts to approximately $2.26B of medical expenses attributable to dampness and mold.

#### 4.2.4. Value of Sick Days Lost to Allergic Rhinitis

Bhattacharyya [22] estimates that on average, each working adult AR patient takes approximately 0.6 sick work absent days annually, which would amount to 0.84 overall sick days including nonworkdays. Assuming the same proportion for working and nonworking patients results in a total 3.01 million sick days valued at $79.05 million for adult workers, adult nonworkers, and children. Children account for 0.45 sick days and 0.24 missed schooldays.

#### 4.2.5. Value of Reduced Productivity

Bhattacharyya [22] estimated an incremental 42% risk of having functional work limitations when compared to non-AR patients. Data from Mitchell and Bates [23] suggest that each working adult having no more than one medical condition has approximately 5.3 “unproductive” days of work each year, which would amount to 7.5 total unproductive days, including weekend days. For the purpose of this paper, it is assumed that each “unproductive day” amounts to a 25% productivity loss (base case), or 0.25 equivalent productive days. Using the 7.5 unproductive days per person as the baseline for the 42% incremental risk for AR patients results in a total of 2.82 million equivalent lost days because of reduced productivity valued at $222.88 million attributable to dampness and mold. Children's portion accounts for 0.30 million equivalent missed schooldays lost to reduced productivity (reduced learning).

#### 4.2.6. Patient Care of Sick Children

It is assumed that a sick day from AR for an adult patient means that they stay home with activity and social restrictions but can take care of themselves, so there are no lost days calculated for family or friends. However, it is assumed that each sick child day would account for an equal number of adult days lost in their care. Of the 0.45 million adult days lost, 0.29 million are workers and 0.16 million are nonworkers, whose combined time lost totals $33.53 million.

#### 4.2.7. Total Base Case Cost for Allergic Rhinitis

The total COI cost for allergic rhinitis was $2.257 billion for direct medical expense costs and $333.53 million for indirect costs, for a total of $2.593 billion attributable to dampness and mold under base case assumptions.

### 4.3. Base Case for Asthma and Acute Bronchitis Hospitalization

Table 3 provides a summary of base case costs for asthma and acute bronchitis hospitalization associated with exposure to dampness and mold.

#### 4.3.1. Proportion of Asthma and Acute Bronchitis Hospitalization Cases due to Dampness and Mold

Assuming 35% prevalence for dampness and mold means that 16% of asthma cases and 14% of bronchitis cases can be attributed to dampness and mold exposure.

#### 4.3.2. Medical Expenses Related to Hospitalization and Recovery

For asthma and acute bronchitis, all costs that are estimated are related to a hospital stay. They include direct hospital charges, charges for other services while at the hospital but not directly charged by the hospital, and follow-up costs for doctor visits and medications during recovery. Data on the number of hospitalization cases (adjusted to reflect the 2014 population) and average length of stay are from CDC [25]. Data for acute bronchitis are taken from the category that includes bronchitis and bronchiolitis.

An estimate of $10,525 per hospital stay is used for both asthma and acute bronchitis. This value is the inflation adjusted average cost for all hospitalization cases [26]. Estimated direct hospital charges attributable to dampness and mold were $768 million for asthma and $263 million for acute bronchitis.

Thayer et al. [2] provides information on extra expenses during a hospital stay for chronic respiratory and acute respiratory illnesses that are separately billed by the providers and not charged by the hospital, as well as medical expenses during recovery from the hospital. It is assumed that such expenses would be proportional to length of stay, which is 3.6 days for asthma and 2.9 days for acute bronchitis [25]. Adjusting for inflation, the resulting attributable extra costs are $21.40 million for asthma and $4.70 million for acute bronchitis, leaving a total hospital stay expense of $789.40 million and $267.50 million, respectively.

Thayer et al. [2] provides similar data on the number of days and medical expenses during recovery, where recovery time is generally several times longer than a hospital stay. It is estimated here to be 17 days for asthma and 19 days for acute bronchitis. Assuming that expenses in recovery are also proportional to length of recovery time, attributable medical expenses during recovery are estimated to be $90.3 million for asthma and $19.2 million for acute bronchitis.

#### 4.3.3. Value of Sick Patient Days during Hospital and Recovery

Sick absentee days for patients and time taken by family and friends are calculated and valued for workers and nonworkers separately. For patients, the days hospitalized and days in recovery provided the number of patient sick days.

There were approximately 0.87 million worker and 0.50 million nonworker patient sick days for asthma during hospitalization and recovery attributable to dampness and mold. Comparable figures for acute bronchitis were 0.30 million worker and 0.18 million nonworker sick days. The economic value of such losses was $78.74 million for working asthma patients, $31.83 million for nonworking asthma patients, $29.26 million for working acute bronchitis patients, and $12.57 nonworking acute bronchitis patients.

Computations for sick children were the same as for adults. Accordingly, the number of sick days for children was 0.24 million for asthma and 0.09 million for acute bronchitis, leading to a value of $7.70 million for asthma and $3.04 million for acute bronchitis. Of those sick days, 0.08 million and 0.03 million were missed schooldays for asthma and acute bronchitis, respectively.

#### 4.3.4. Value of Days Lost by Family and Friends

Thayer et al. [2] provide information on time spent by family and friends during adult hospitalization and recovery for both chronic respiratory and acute respiratory hospitalization. Using ratios of time spent by family and friends to patient sick days, it is estimated that approximately 0.12 million worker and 0.07 million nonworker days are spent by family and friends for asthmatic adults, and 0.03 million worker and 0.02 million nonworker days are spent for acute bronchitis adult patients. The economic value of such losses was $11.73 million for workers and $4.70 million nonworkers for asthma. Corresponding values for acute bronchitis were $2.49 million and $1.21 million.

It was assumed that one adult day was lost in caring for a child for every day of illness. Accordingly, there were 0.24 million adults in child care for asthma children and 0.09 million for children with acute bronchitis. For children with asthma, the value for working adults was $14.80 million and for nonworking adults was 5.98 million. Corresponding values for acute bronchitis were $5.55 million and $2.24 million.

#### 4.3.5. Attributable COI Total Cost for Asthma and Acute Bronchitis Hospitalization

Given the above, the total estimated medical expenses and COI wage-related losses attributable to dampness and mold exposure result in COI economic costs of $1.035 billion $343 million for asthma and acute bronchitis hospitalization, respectively.

### 4.4. WTP for Asthma and Acute Bronchitis Hospitalization and Recovery

Johnson et al. [16] used a stated preference analysis and combined two traditional formats (graded-pair and discrete-choice) of persons' willingness to pay to avoid episodes of illnesses described in terms of type of symptom and intensity, its duration, and activity limitations. For this analysis, we used the symptom category of “breath” that includes coughing, wheezing, and shortness of breath; a limiting activity category of “in hospital”; and a recovery category of “at home,” meaning that the affected individuals cannot go to work or school but can take care of themselves. The study was done with Canadian subjects and in Canadian dollars. Interpolating for the length of stay in days for hospital and recovery, respectively, and adjusting for both inflation and a historical exchange rate of 0.8 US dollars per Canadian dollars, the WTP value per hospital stay (hospital episode) and recovery period (recovery episode) are presented in Table 4.

The WTP values to avoid a single hospital asthma episode and single asthma hospital and recovery period are estimated to be $728 and $799, respectively, with comparable values of $676 and $806 for acute bronchitis. Applying these values for all asthma hospital and recovery episodes attributable to dampness and mold results in a willingness to pay value of $52.6 million and $62.0 million for a total of $114.5 million. For acute bronchitis, the WTP values were $18.64 million and $19.62 million, for a total of $38.3 million. Table 4 breaks these values down by worker, nonworker, and children based on the number of cases for each.

### 4.5. Sensitivity Analysis

Different calculation scenarios were developed to assess sensitivity of results to different assumptions. Each was applied to the cost estimates in Tables 2, 3, and 4. Results are presented in Table 5.

Three basic options and one multiple option were developed:

- Option 1 uses just base case COI costs as presented in the paper.
- Option 2 adds WTP for pain and suffering to the COI costs for adult patient workers,and substitutes WTP for COI values for all patient nonworkers and children. This does not apply to AR patients (Section 3).
- Option 3 adds WTP to the COI values for patient workers but uses COI only for patient nonworkers and children. This does not apply to AR patients (Section 3).
- Option 4 uses Option 3 as a base but recalculates values incrementally adding the following changes: (a) uses full median wage rather than portions thereof for all nonworkers and children, (b) assigns values for weekend days the same as workdays, (c) uses average rather than median wage, and (d) assumes a 47% prevalence for dampness and mold rather than 35% to calculate AF.

The incremental changes (a)–(d) in Option 4 above were used against the base calculation (Option 1). For AR, two categories, AR (gov) and AR (alt), are used for completeness. AR (gov) is the base case previously presented, while AR (alt) uses direct medical costs estimated by Bhattacharyya [21]. Option 4(d) reflects an assumption of 47% rather than 35% prevalence of dampness and mold. That increases the estimate of the attributable fraction from 15% to 19% for allergic rhinitis, 16% to 21% for asthma, and 14% to 17% for acute bronchitis.

#### 4.5.1. Range of Potential Cost Estimates

In a low but reasonable cost estimate, medical costs might include just direct medical charges, while indirect costs might include the value of lost workdays due to illness for working patients only. These are not uncommon assumptions not previously considered. Such a low cost estimate is given in Table 6 and is lower than the base case that also accounts for lost days for nonworking patients, for family and friends and for children. In addition, for the cost of hospitalization, neither extra hospital charges nor costs during recovery are included. Losses for nonworking patients or family and friends or from reduced productivity are also not included. As a result, this low cost estimate is 11%, 25%, and 21% below the base case (Option 1) estimate for AR, asthma, and bronchitis, respectively.

Comparing Table 6 with Table 5 further demonstrates how a wide range of cost estimates is possible. Comparing the two tables, the range of total economic cost estimates for AR is $2.3 billion to $4.0 billion or to $4.8 billion for the alternative estimate. Similarly for asthma and bronchitis hospitalization, the range of total cost is $781 million to $1.7 billion for asthma and $270 million to $575 million for bronchitis. This shows an approximate doubling, or more, of estimates from the lowest to the highest.

Table 7 examines the impact on the ratio of indirect to direct costs under each option. It demonstrates that the importance of indirect costs relative to direct costs can depend significantly on the assumptions chosen about the value of time lost for workers, nonworkers, and children for both patients and nonpatients involved. This ratio generally increased by about 25% from the base case to Option 4(c) in all diseases.

Indirect costs constituted 13–16% of total costs in the base case and 28–35% in Options 4(c) & 4(d) where full wage and time assumptions were used. Time spent by family and friends, including caring for sick children, accounts for 8–10% of indirect costs for AR, 10–15% for asthma, and 6-7% for bronchitis. Unproductive days accounted for approximately 65% of indirect costs for AR. WTP estimates for nonworking adults and children fell between COI estimates using partial wage assumptions and full wage assumptions. Finally, changing assumptions of dampness and mold prevalence, from 35% to 47%, had a large impact on the number of disease cases and costs, changing all estimates by about 28%. Table 7 shows that indirect cost can more than double as a percent of total cost depending on the valuation methods and what cost elements are included. This is consistent with other studies [2, 13].

### 4.6. Final Estimates of the Full Annual Cost of Each Disease

The method described in Option 4(b) of the sensitivity analysis is the method used for the final cost estimates. In this option, indirect costs account for burdens of family and friends as well as the patient. It valuates all patient sick days, not just missed workdays and schooldays, because all sick days are burdensome. It uses the median wage, which is lower than the average wage, because it is more representative. It assumes that the prevalence of dampness and mold is 35%, rather than 47%, because 35% represents the center of current estimates from a broad range of climates. In addition, since 5.3% of the population does not get needed health care and approximately double that amount does not have health insurance [27], a conservative 6% increment is used in the final estimates to account for the underutilization of health care services.

Final cost estimates from all causes, not limited to dampness and mold, are also provided. These are computed by dividing the illness cost from dampness and mold by its corresponding attributable fraction. Conversions from attributable costs to the costs for all cases of illness reflect this same relationship.

#### 4.6.1. Allergic Rhinitis

The morbidity costs for allergic rhinitis for Option 4(b) is $2.9 (2.3–5.1) billion (government estimate) and $3.5 (2.3–5.1) billion (Bhattacharyya [21] estimate). The latter estimate is chosen because it includes a controlled attribution of costs of comorbid illnesses. The 6% increment to account for underutilization of health care results in a total cost estimate of $3.7 (2.3–5.4) billion for allergic rhinitis attributed to dampness and mold. The corresponding value for all allergic rhinitis cases would be $24.8 billion. This range reflects the range established in the sensitivity analysis plus the 6% increment.

#### 4.6.2. Acute Bronchitis

The morbidity costs for acute bronchitis hospitalization due to dampness and mold in Option 4(b) of the sensitivity analysis is $483.3 million. That would correspond to an estimate of $3.5 billion for hospitalization of all acute bronchitis cases, not limited to dampness and mold. A WTP estimate provided by EPA [1] for all nonhospitalized cases is $584 per incidence when adjusted to 2014$. Assuming one incidence per nonhospitalized case, and accounting for the 6% of underutilization in health care, the total cost for all acute bronchitis patients would be $13.5 billion. The estimated cost for acute bronchitis attributable to dampness and mold is thus $1.9 (1.1–2.3) billion. This range in costs is proportional to the range for just hospitalization established in the sensitivity analysis.

#### 4.6.3. Asthma

Jang et al. [17] provide an estimate of incremental annual expenditures (direct costs) for health care services in the US, plus the number of missed workdays and missed schooldays (indirect costs) per asthmatic. Adjusted to 2014, the estimate is $71.7 billion for direct costs, plus 6.8 missed workdays, and 4.2 missed schooldays per asthmatic.

When missed workdays and schooldays are converted to all sick days and when valuation by methods in Option 4(b) are applied, indirect cost estimates come out to $770 and $670 per asthmatic adult and child, respectively, or approximately $19.6 billion. This brings the total of direct and indirect costs to $91.3 billion. Accounting for the 6% of underutilized health care services, the total cost for all cases of asthma would be approximately $94.5 billion. The total morbidity cost attributed to dampness and mold is thus $15.1 (9.4–20.6) billion. This range in costs is proportional to the range for just hospitalization established in the sensitivity analysis.

Jang et al. [17] did not estimate the value of asthma mortality. A similar study by Barnett and Nurmagambetov [18] provided a mortality estimate of approximately 3,647 deaths (adjusted to 2014 population). The authors use the COI method for discounted rest-of-expected-life earnings valued at an average of $686,000 per asthma death (2014$). In this calculation, since most asthma deaths occur in persons above 65 years old, the discounted rest-of-expected-life earnings are extremely low and unlikely represent the full cost to society.

EPA's WTP value of a single premature death is approximately $8 million (2014$) [1], but this estimate does not account for the age at premature death. To do that for asthma cases, it is noted that the $8 million estimate is mostly based on wage differential studies of midaged adults. The average COI value of a premature death for the highest 3 mid-working-age adults (15–44 years) is approximately $1.7 million per death [18]. Thus, the WTP computed for midaged adults is over 4.5 times higher than the COI estimate for midaged adults in [18]. Multiplying the age-adjusted value of $686,000 [18] by 4.5 provides a rough approximation of an age-adjusted WTP value of a single mortality for asthma of $3.1 million per death or $10.8 billion total for all asthma cases. The estimate for asthma mortality due to exposure to dampness and mold is thus approximately 584 annual deaths valued at $1.7 (0.4–4.5) billion. This range reflects the different options for valuing premature death.

## 5. Conclusion

Meta-analysis results suggest that 15–20% of allergic rhinitis, acute bronchitis, and asthma costs can be attributed to an indoor dampness and mold prevalence of 35%. The full cost of these diseases is estimated, while the range of costs is calculated through a sensitivity analysis of various costing assumptions. The final cost estimates are designed to account for costs that are commonly excluded or undervalued in traditional applications of the COI method.

Conceptually, WTP fully captures the full cost to society. However, since WTP values are difficult to compute and rarely available, COI values are most commonly used. Including a fuller range of costs in COI estimates makes it more likely that they will better reflect the true cost to society.

The method used in this paper to estimate the full costs for these diseases can be applied to other health risks generally and to specific risks associated with indoor air quality. Further research could help determine whether expanding cost considerations in COI estimates compares favorably with WTP estimates and in what circumstances they differ. This would help clarify how we understand and apply each method and whether the differences are significant.

Using a more inclusive approach, the annual costs attributable to dampness and mold are estimated to be $3.7 (2.3–4.7) billion for allergic rhinitis, $1.9 (1.1–2.3) billion for acute bronchitis, $15.1 (9.4–20.6) billion for asthma morbidity, and $1.7 (0.4–4.5) billion for asthma mortality. Given the ambiguity of the specific causal agents that may account for the associations found between illnesses and damp and moldy conditions, these estimates must be considered preliminary. More research is needed to determine specific agents causing the illnesses. This would help guide mitigation methods in damp and moldy buildings in order to insure that mitigation is effective in reducing the risk. Further research on the effectiveness of mitigation would also be valuable.

The studies on which these estimates are based are limited to homes, where people spend 70–90% of their time, but do not include the total exposure from other environments. Similar associations have been found in schools especially but also in office environments [13]. Still, little is known about the effect that length of exposure to dampness and mold has on health risks. Total exposure research on dampness and mold would also be helpful. Such research would also benefit from what specific causal agents need to be measured in order to best characterize exposure.

## Figures and Tables

**Table 1:** Meta-analysis results for risk and attributable fractions of risks of selected diseases from indoor dampness and mold exposure.

Reference	Disease	Disease prevalence	Risk factor	OR (95% CI)	AF
Jaakkola et al. [8]	Allergic rhinitis	0.075^a^	Visible mold	1.51 (1.39–1.64)	0.15

Fisk et al. [9]	Current asthma	0.082^a^	Dampness &/or mold	1.56 (1.30–1.86)	0.16

Fisk et al. [10]	Acute bronchitis	0.050^b^	Dampness &/or mold	1.45 (1.31–1.59)	0.14

^a^CDC [14] and ^b^Worrall [15].

**Table 2:** Number of AR patients attributable to dampness and mold and base case COI monetary value of impacts in 2014.

	Number million	Monetary value million $
AR patients	3.59	

Direct medical expenses ($629.69/patient)		**2,257.33**

Patient sick days		
Working adults	1.63	52.73
Nonworking adults	0.93	21.19
Children	0.45	5.13
Total	3.01	**79.05**
Missed schooldays^*∗*^	0.24	

Patient reduced productivity (equivalent days)		
Working adults	4.59	148.68
Nonworking adults	2.63	59.75
Children	1.27	14.45
Total	8.49	**222.88**
Missed schooldays^*∗*^	0.68	

Adult lost days for care for sick children		
Working adults	0.29	27.92
Nonworking adults	0.16	5.61
Total	0.45	**33.53**

Total direct medical expenses		2,257.33

Days & COI values for wage related losses	11.95	335.46

*Total COI economic cost*		*2,592.79*

^*∗*^Schooldays portion of school-age children days.

**Table 3:** Number of asthma and acute bronchitis hospitalization events & economic value attributable to dampness and mold exposure (2014).

	Asthma		Acute bronchitis	
	Number millions	Value mill $	Number millions	Value mill $
Medical costs in hospital				
Days	0.26		0.07	
Direct charges		768.00		263.00
Added charges		21.40		4.50
Total charges		789.40		267.50

Medical costs recovery (at home)				
Days	1.34		0.49	
Costs		90.30		19.20

Patient sick days: adult worker				
In hospital	0.14		0.04	
In recovery	0.72		0.26	
Total adult worker	0.87	78.74	0.30	29.26

Patient sick days: adult nonworker				
In hospital	0.08		0.03	
In recovery	0.42		0.15	
Total adult nonworker	0.50	31.83	0.18	12.57

Patient sick days: children				
In hospital	0.04		0.01	
In recovery	0.20		0.07	
Total	0.24	7.70	0.09	3.04
Missed schooldays^*∗*^	0.08		0.03	

Family & friends: adult worker				
In hospital	0.06		0.01	
In recovery	0.06		0.01	
Total adult worker	0.12	11.73	0.03	2.49

Family & friends: nonworker				
In hospital	0.03		0.01	
In recovery	0.04		0.01	
Total adult nonworker	0.07	4.70	0.02	1.21

Family & friends child care:				
Workers	0.15	14.80	0.06	5.55
Nonworkers	0.09	5.98	0.03	2.24
Total	0.24		0.09	

*Total direct medical expenses*		*879.7*		*286.7*

*Total COI wage related costs*		*155.48*		*56.36*

*Total COI economic costs*		*1035.18*		*343.06*

^*∗*^Missed schooldays are a portion of children sick days.

**Table 4:** WTP for patients of asthma and acute bronchitis by working status and children.

	Number of cases (million)	Value for hospital (million $)	Value of recovery (million $)	Total value (million $)
Asthma				
Workers	0.04	28.29	33.31	
Nonworkers	0.02	16.17	19.43	
Children	0.01	8.08	9.25	
Total	**0.07**	**52.55**	**61.99**	**114.54**

Acute bronchitis	0.01	9.32	10.63	
Workers	0.01	6.99	6.13	
Nonworkers	0.00	2.33	2.86	
Children	0.01	0.00	0.00	
Total	**0.03**	**18.64**	**19.62**	**38.26**

**Table 5:** Costs under optional calculation scenarios.

	Option 1 (million $)	Option 2 (million $)	Option 3 (million $)	(Million $)
AR (gov)	**2592.75**	NA	NA	—
AR (alt)	**3194.74**	NA	NA	—
Asthma hosp	**1,035.18**	1,066.18	1,096.78	—
Acute bronch hosp	**343.06**	365.71	363.01	—

Option 4	(a)	(b)	(c)	(d)

AR (gov)	2,738.08	2,930.38	3,151.71	**3992.17 **
AR (alt)	3340.03	3532.33	3753.66	**4756.64**
Asthma	1159.76	1248.20	1349.98	**1709.97**
Acute bronch	476.45	483.26	507.62	**575.30 **

**Table 6:** Low estimate: base case with limited cost categories.

	Allergic rhinitis (million $)	Asthma hosp (million $)	Acute bronch hosp (million $)
Direct medical	2,257.33	768.00	263.00
Indirect	42.80	12.67	3.90

*Total*	*2300.14*	*780.67*	*269.90*

**Table 7:** Indirect cost as a percent of total cost.

	AR (gov)	AR (alt)	Asthma hospital	Acute bronchitis hospital
Base (opt 1)	15%	12%	18%	20%
Full WTP (opt 2)	NA	NA	21%	28%
Partial WTP (opt 3)	NA	NA	25%	27%
Full med wage (opt 4(a))	21%	17%	32%	28%
All days (opt 4(b))	30%	24%	42%	35%
Avg wage (opt 4(c))	40%	31%	53%	53%
47% prevalence of DM (opt 4(d))	40%	31%	53%	53%

## References

[1] US Environmental Protection Agency (EPA) (2011). Benefits and costs of the clean air 1990–2010. *Final Report*.

[2] Thayer M. A., Chestnut L. G., Lazo J. K., Van Den Eeden S. K.

[3] Bornehag C.-G., Blomquist G., Gyntelberg F. (2001). Dampness and buildings and health. nordic interdisciplinary review of the scientific evidence on associations between exposure to “Dampness” in Buildings and Health Effects (NORDDAMP). *Indoor Air*.

[4] Bornehag C. G., Sundell J., Bonini S. (2004). Dampness in buildings as a risk factor for health effects, EUROEXPO: a multidisciplinary review of the literature (1998–2000) on dampness and mite exposure in buildings and health effects. *Indoor Air*.

[5] Clark N. M., Ammann H. M., Brunedreef B. (2004). *Damp Indoor Spaces and Health*.

[6] WHO (2009). *WHO Guidelines for Indoor Air Quality Dampness and Mold*.

[7] Mendell M. J., Mirer A. G., Cheung K., Tong M., Douwes J. (2011). Respiratory and allergic health effects of dampness, mold, and dampness-related agents: a review of the epidemiologic evidence. *Environmental Health Perspectives*.

[8] Jaakkola M. S., Quansah R., Hugg T. T., Heikkinen S. A. M., Jaakkola J. J. K. (2013). Association of indoor dampness and molds with rhinitis risk: a systematic review and meta-analysis. *Journal of Allergy and Clinical Immunology*.

[9] Fisk W. J., Lei-Gomez Q., Mendell M. J. (2007). Meta-analyses of the associations of respiratory health effects with dampness and mold in homes. *Indoor Air*.

[10] Fisk W. J., Eliseeva E. A., Mendell M. J. (2010). Association of residential dampness and mold with respiratory tract infections and bronchitis: a meta-analysis. *Environmental Health: A Global Access Science Source*.

[11] Antova T., Pattenden S., Brunekreef B. (2008). Exposure to indoor mould and children's respiratory health in the PATY study. *Journal of Epidemiology and Community Health*.

[12] Quansah R., Jaakkola M., Hugg T. T., Heikkinen S. A. M., Jaakkola J. J. K. (2012). Residential dampness and molds and the risk of developing asthma: a systematic review and meta-analysis. *PLoS ONE*.

[13] Mudarri D., Fisk W. J. (2007). Public health and economic impact of dampness and mold. *Indoor Air*.

[14] CDC Summary of Health Statistics for Us. Adults: National Health Interview Survey. http://www.cdc.gov/nchs/data/series/sr_10/sr10_260.pdf.

[15] Worrall G. (2008). Acute bronchitis. *Canadian Family Physician*.

[16] Johnson F. R., Banzhaf M. R., Desvousges W. H. (2000). Willingness to pay for improved respiratory and cardiovascular health: a multiple-format, stated-preference approach. *Health Economics*.

[17] Jang J., Chan K. C. G., Huang H., Sullivan S. D. (2013). Trends in cost and outcomes among adult and pediatric patients with asthma: 2000–2009. *Annals of Allergy, Asthma and Immunology*.

[18] Barnett S. B. L., Nurmagambetov T. A. (2011). Costs of asthma in the United States: 2002–2007. *Journal of Allergy and Clinical Immunology*.

[19] BLS National Occupational Employment and Wage Estimates: United States. http://www.bls.gov/oes/current/oes_nat.htm.

[20] Soni A. Allergic Rhinitis: Trends in Use and Expenditures, 2000 and 2005.

[21] Bhattacharyya N. (2011). Incremental healthcare utilization and expenditures for allergic rhinitis in the United States. *Laryngoscope*.

[22] Bhattacharyya N. (2012). Functional limitations and workdays lost associated with chronic rhinosinusitis and allergic rhinitis. *American Journal of Rhinology and Allergy*.

[23] Mitchell R. J., Bates P. (2011). Measuring health-related productivity loss. *Population Health Management*.

[24] United States Census Bureau (2016). *Monthly Population Estimates for the United States: April 1, 2010 to December 1, 2015*.

[25] CDC National Hospital Discharge Survey. http://www.cdc.gov/nchs/nhds/nhds_tables.htm#number.

[26] Pfuntner A., Wier L. M., Steiner C. Costs for Hospital Stays in the United States, 2010.

[27] Ward B. W., Clarke T. C., Freeman G., Schiller J. S. Early Release of Selected Estimates Based on Data From the 2014 National Health Interview Survey. http://www.cdc.gov/nchs/data/nhis/earlyrelease/earlyrelease201506.pdf.

